# Acceptance and hesitancy of COVID-19 vaccination among Saudi pregnant women

**DOI:** 10.3389/fgwh.2024.1336766

**Published:** 2024-01-26

**Authors:** Sereen Hawsawi, Abeer Orabi, Hend Alnajjar

**Affiliations:** ^1^ Jeddah East Hospital, Jeddah, Saudi Arabia; ^2^ Nursing Department, College of Nursing, King Saud bin Abdulaziz University for Health Sciences, Jeddah, Saudi Arabia; ^3^ King Abdullah International Medical Research Center, Jeddah, Saudi Arabia

**Keywords:** COVID-19, pandemic, vaccine hesitancy, acceptance, pregnant women, Saudi Arabia

## Abstract

**Introduction:**

Since the COVID-19 pandemic started, it has significantly impacted health systems and societies worldwide. Pregnancy increases the risk of severe illness when associated with COVID-19. Pregnant women are likely to experience COVID-19-related pregnancy complications however many of them were hesitant to receive the vaccine. This study aimed to assess the acceptance and hesitancy of COVID-19 vaccination among pregnant women in Jeddah.

**Methods:**

The present study utilized a descriptive cross-sectional research design to include pregnant women through convenience sampling at the obstetrics and gynecology outpatient department and antenatal ward at King Abdul-Aziz Medical City, Jeddah. Data was collected from August to October 2022 using a structured questionnaire.

**Results:**

Approximately one-third of the 264 participants (36.4%) strongly disagreed that they had developed immunity against COVID-19. More than half of them (61% and 66.3% respectively) had heard that the vaccine was unsafe for them and their babies during pregnancy, and it would contain the COVID-19 virus. However, half of them 50% agreed that vaccination would ease precautionary measures. A high acceptance score toward COVID-19 vaccination was observed among pregnant women working in the health sector. The most frequently reported reason for vaccination refusal was the belief that it was unsafe for the mother and her baby during pregnancy.

**Discussion:**

Most of the participants perceived their susceptibility to COVID-19 infection as low and were hesitant to take the vaccine because of their worry about its effectiveness. However, they indicated a willingness to take the vaccine if it was mandatory and if they received adequate information about it. These findings correspond with previous studies conducted in Saudi Arabia that explore the reluctance of pregnant and breastfeeding women to receive COVID-19 vaccination.

## 1 Introduction

The COVID-19 pandemic was initiated by the severe acute respiratory syndrome coronavirus 2 (SARS-CoV-2) and was first documented in Wuhan, China, at the end of 2019 (1). Globally, it has led to over 496 million cases and approximately 6 million deaths (2). In Saudi Arabia, up until 2022, the Ministry of Health reported a total of 752,078 confirmed COVID-19 cases and 9,060 deaths (3). It is important to note that pregnancy is a significant factor that increases the risk of severe illness when associated with COVID-19 infection (4). This heightened vulnerability can be attributed to transient immunosuppression and physiological changes in the respiratory and cardiovascular systems during pregnancy (5–8). A study in Saudi Arabia from March to October 2020 revealed a prevalence rate of 4.2% among pregnant women. Notably, 62% of these women were asymptomatic at diagnosis, and there was one reported death due to respiratory failure in this group (9).

Pregnant women are more likely to experience COVID-19-related pregnancy complications, such as preterm deliveries, neonatal intensive care unit admissions, and even mortality when infected with the virus (10). Several studies have demonstrated that pregnant women are highly susceptible to the SARS-CoV-2 virus, resulting in severe symptoms and worsened pregnancy outcomes (4, 11, 12). Furthermore, research from China and the United States has indicated a comparable risk of severity among pregnant patients when compared to the general population (13–15).

Symptoms of COVID-19 in pregnant women encompass fever, fatigue, dyspnea, and cough, with severity ranging from mild to severe instances, potentially progressing to acute respiratory distress syndrome, which can adversely affect the gastrointestinal, cardiovascular, and neurological systems, leading to multiple organ failures (16). Notably, Breslin et al. (14) reported two cases of maternal mortality, while Liu et al. (11) described several organ dysfunction syndromes, including acute respiratory distress syndrome resulting in fetal loss. Additional COVID-19-related pregnancy comorbidities include maternal hyperglycemia, hypertension, pneumonia, and venous thromboembolism (17, 18). Furthermore, there have been reports of increased intensive care unit admissions, mechanical ventilation, caesarean sections, intubations, miscarriages, preeclampsia, coagulopathy, and premature rupture of membranes among pregnant women infected with COVID-19 (19). Infant complications resulting from COVID-19 infection during pregnancy include prematurity, low birthweight, intrauterine transmission of infection, stillbirth, and fetal growth restriction (20–24).

In response to the negative consequences of the pandemic, governments have implemented preventive measures to curb the virus's spread. These measures encompass working from home, school closures, and the suspension of recreational and social activities. Preventive strategies such as hand washing, social distancing, and COVID-19 vaccination have been employed (25). The WHO has introduced The Global COVID-19 Vaccination Strategy to minimize deaths, reduce severe diseases, and mitigate the impact on healthcare systems, while facilitating the resumption of socioeconomic activities and reducing the risk of new variants. This is contingent on achieving widespread immunity by fully vaccinating at least 70% of the world's population, which includes most adults, adolescents, and individuals at risk for severe disease, including pregnant women (26). A study from Brazil highlighted the dynamics of COVID-19 vaccine uptake among pregnant women, showing an uptake rate of 53%. Factors influencing vaccine uptake included age, more antenatal care appointments, and a history of child loss (27).

Despite the recommendations of the Saudi Ministry of Health, which encouraged receiving COVID-19 vaccines as a preventive strategy for both males and females, including the elderly and pregnant women (3), many pregnant women have expressed vaccine hesitancy due to concerns about its safety during pregnancy and lactation. This hesitancy has hindered the vaccination strategy (10, 28). Factors influencing COVID-19 vaccine acceptance among pregnant women were being mandatory vaccination in many countries (29), to unclear information regarding the advantages, safety, and effectiveness of the vaccine, as well as fears of side effects for both the pregnant woman and her fetus, preference for other methods of COVID-19 prevention, and concerns that the vaccine could lead to COVID-19 infection (10, 30).

Nurses and midwives can play a significant role in preventing the spread of COVID-19 among pregnant women by assessing the factors contributing to vaccine hesitancy, outlining strategies to address these concerns, promoting preventive measures, and adhering to strict hygiene and isolation protocols in maternity wards. Additionally, they should provide comprehensive care to pregnant women infected with COVID-19, including monitoring vital signs, maintaining electrolyte balance, preventing dehydration, and offering psychological support (31, 32). Furthermore, they should engage in postnatal education and enlightenment programs (33).

In the Middle East, there is a paucity of studies concerning acceptance and hesitancy about COVID-19 vaccination among pregnant women, with most research focused on the adult general population and healthcare workers. However, a study conducted in Saudi Arabia focused on examining pregnant women's acceptance and hesitancy, revealing that pregnant women and those planning to become pregnant had a moderate score in terms of their perceptions of COVID-19 vaccination, reluctance, perceived benefits, and reasons for action (34). Previous studies have highlighted significant variations in COVID-19 vaccination acceptance rates and hesitancy among pregnant women in different countries. Therefore, it is essential to assess the hesitancy and acceptance of COVID-19 vaccines among pregnant women in Saudi Arabia to understand the factors influencing their decisions and design programs that empower women to make informed choices based on current scientific evidence. This approach will not only promote pandemic prevention but also enhance women's healthcare in general.

## 2 Materials and methods

### 2.1 Study design and setting

A descriptive cross-sectional design was employed in this study to assess the acceptance and hesitancy of COVID-19 vaccination among Saudi pregnant women. The study was conducted at the antenatal and gynecology ward (Ward 1), as well as the obstetrics and gynecology outpatient department (OPD), in King Abdulaziz Medical City, Western Region.

### 2.2 Sample and sampling

The study included 264 pregnant women equally recruited from the ward and the OPD, using convenience sampling. This nonprobability sampling method relies on readily available data sources. The inclusion criteria were Saudi women aged 18 years or older, who were able to read and write, and willing to participate in the study. The sample size was determined using G-Power online calculator, considering the average monthly admissions in the ward and the antenatal care visits at the outpatient department, with a confidence level of 95% and a margin of error of 5%.

### 2.3 Instrument and data collection

Data was collected from August to October 2022 using a self-administered structured questionnaire adapted from previous studies (34, 35). The questionnaire is divided into five sections. The first section gathers demographic characteristics like age, education, occupation, medical history, gestational age, number of pregnancies, children, previous COVID-19 infection, and vaccination status. The second section assesses perceptions of COVID-19’s severity and susceptibility, including personal experiences and perceived risk in Saudi Arabia. The third section explores hesitancy towards the COVID-19 vaccine, addressing concerns about safety, allergies, misinformation, and vaccine components. The fourth section delves into the perceived benefits of vaccination, such as easing precautionary measures and protecting against COVID-19 complications. The final section focuses on reasons for receiving the COVID-19 vaccine, including mandatory policies, public acceptance, and the need for adequate information. Each item was scored using a five-item Likert scale ranging from 1 (Strongly disagree) to 5 (Strongly agree). The questionnaire was developed in English, then translated into simple Arabic using competent services then back-translated into English and revised accordingly to ensure that the intended meaning of the items is kept.

The validity of the questionnaire was confirmed by a panel of experts in the nursing and obstetrics fields who rated each item in terms of its relevance to the underlying construct and clarity. Then I-CVI and S-CVI/Ave were computed. To confirm the reliability of the questionnaire, the degree of error was assessed through pilot testing to make sure it was sufficiently comprehensive and well-structured. The reliability results through Cronbach's Alpha testing for Likert scales was (0.824) which was satisfactory. A pilot study was conducted by distributing the questionnaire to 53 pregnant women (20% of the sample size) who met the inclusion criteria to assess its completeness and applicability. No modifications were needed and collected data for piloting was not a part of the whole study.

### 2.4 Data analysis

The collected data was coded for entry and analyzed using statistical software (SPSS) version 28 for Windows. Descriptive statistics, such as frequencies, percentages, means, and standard deviations, were used to present the data. Inferential statistics, including ANOVA test and Pearson correlation, were employed to examine the association and significance of comparisons between study variables. A *P*-value of ≤0.05 was considered statistically significant.

### 2.5 Ethical consideration

The study was conducted with the approval of the Research Unit at the College of Nursing, King Abdullah International Medical Research Center, and the Institutional Review Board at King Abdulaziz Medical City-Western Region (IRB Approval No: IRB/1218/22). The researcher explained the study's aim and obtained informed consent from each participant; also, they were assured of the voluntary nature of their participation, with the right to discontinue or refuse to participate at any time. Confidentiality and privacy of responses were maintained.

## 3 Results

**Figure 1:**
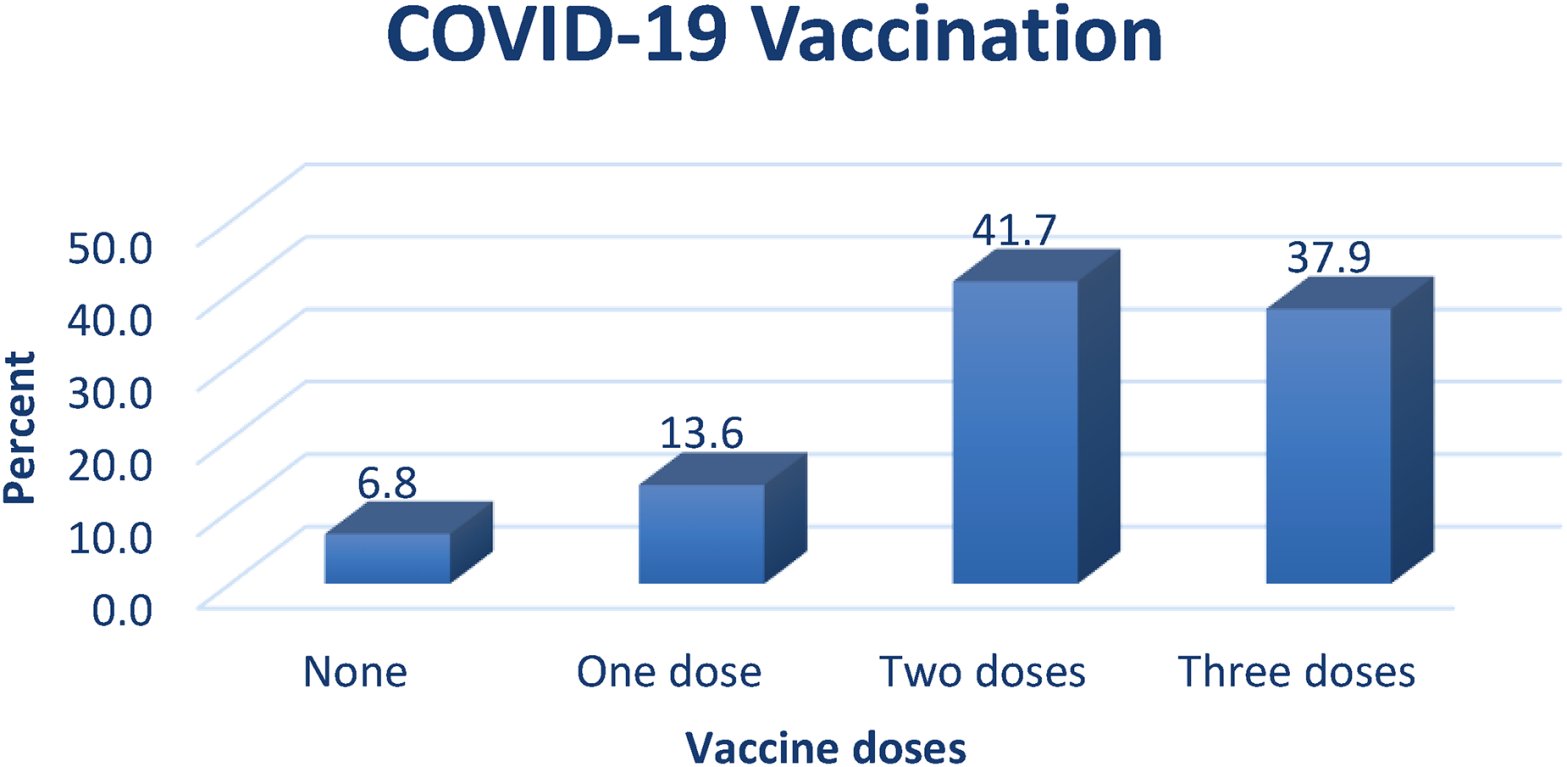
COVID-19 vaccination among the participants.

Among the 264 women surveyed with a response rate of 100%, equally recruited from the ward and the OPD, 45.1% were less than 30 years old, 43.6% had a high school education, and 65.2% were housewives. Results from the medical history data indicated that over half of the participants 56.1% had no pre-existing medical conditions, while 17.4% had diabetes mellitus. More than half of the participants were in their third trimester of pregnancy, and had 1–3 children 60.2% and 54.9%, respectively. Further, 68.2% of them had not experienced a previous COVID-19 infection (Table 1). Concerning COVID-19 vaccination, 41.7% of the participants had received two vaccine doses of those only 0.4% received during pregnancy while 6.8% had not been vaccinated (Figure 1).

**Table 1:** Basic demographic characteristics of the participants.

Variable	*N* = 264
Age	
<30	119 (45.1)
30–34	59 (22.3)
35–39	51 (19.3)
>40	35 (13.3)
Educational level	
Intermediate and less	33 (12.6)
High school	115 (43.6)
Bachelor	105 (39.7)
Postgraduate	11 (4.1)
Occupation	
Housewife	172 (65.2)
Working in health sector	28 (10.6)
Working outside health sector	64 (24.2)
Medical history	
Hypertension	33 (12.5)
Diabetes mellitus	46 (17.4)
Cardiovascular disease	11 (4.2)
Respiratory disease	11 (4.2)
Gastrointestinal disease	11 (4.2)
Immunological disease	3 (1.1)
Nervous system disease	1 (0.4)
None	148 (56.0)
Gestational age	
1st trimester	19 (7.2)
2nd trimester	86 (32.6)
3rd trimester	159 (60.2)
No. of pregnancies	
1–3	165 (62.5)
4–6	71 (26.9)
>7	28 (10.6)
No. of children	
0	66 (25.0)
1–3	145 (54.9)
4–6	44 (16.7)
7+	9 (3.4)
Previous COVID-19 infection	
No	180 (68.2)
Yes	84 (31.8)

Regarding to perception of COVID-19 severity and susceptibility, more than one-third of the participants 36.4% strongly disagreed that they had developed immunity against COVID-19 infection. Additionally, 32.6% of them strongly disagreed that people they know had experienced a mild form of COVID-19, while 24.6% disagreed that the infection is not serious. On the other hand, almost half 48.9% of the participants agreed that the likelihood of contracting a COVID-19 infection is low in Saudi Arabia (Table 2).

**Table 2:** Perception of COVID-19 severity and susceptibility among the participants.

Item	Strongly disagree		Disagree		Neutral		Agree		Strongly agree	
1. I have had COVID-19 infection and I think I have developed immunity against it.	No.	%	No.	%	No.	%	No.	%	No.	%
	96	36.4	62	23.5	44	16.7	55	20.8	7	2.6
2. All people I know who got COVID-19 infection, got the mild form.	86	32.6	55	20.8	45	17.1	66	25	12	4.5
3. COVID-19 infection is not serious so I do not think I should take the vaccine.	65	24.6	45	17.1	52	19.7	78	29.5	24	9.1
4. The likelihood of getting COVID-19 infection is low in Saudi Arabia.	16	6.1	6	2.3	24	9.1	129	48.8	89	33.7
Scale	Min			Max			Mean			SD
	4.0			20.0			11.6			3.5

Considering hesitancy to receive COVID-19 vaccination, more than half of the participants 61%, 61.7%, and 56.8%, respectively reported that they had heard that the vaccine is unsafe for them and their babies during pregnancy, blood clotting is a common side effect of the vaccine, and they were uncertain about its effectiveness during pregnancy (Table 3).

**Table 3:** Hesitancy to receive COVID-19 vaccination among the participants.

Item	Strongly disagree		Disagree		Neutral		Agree		Strongly agree	
1. I have heard that the vaccine is unsafe for me and my baby during pregnancy, so I do not want to take it.	No.	%	No.	%	No.	%	No.	%	No.	%
	33	12.5	12	4.6	22	8.3	161	61	36	13.6
2. I have food or medications allergy so I cannot take the vaccine	77	29.2	54	20.5	75	28.3	49	18.6	9	3.4
3. I have heard that the vaccine is not safe as it would contain the COVID-19 virus.	15	5.7	10	3.8	30	11.4	175	66.2	34	12.9
4. I have heard that blood clotting is a common side effect of the vaccine.	16	6.2	13	4.9	26	9.8	163	61.7	46	17.4
5. I am not sure of the eligibility and registration process.	75	28.4	38	14.4	71	26.9	60	22.7	20	7.6
6. I am not sure about the effectiveness of the vaccine for pregnant women.	14	5.3	13	4.9	33	12.5	150	56.8	54	20.5
7. The vaccine approval process was fast so its safety was not assessed adequately.	10	3.8	4	1.5	27	10.2	149	56.5	74	28
8. I am not sure about the components of the vaccine.	7	2.7	1	0.4	26	9.8	118	44.7	112	42.4
9. I am very strict to precautions (mask, hand washing, and social distance) so I do not need the vaccine.	8	3	4	1.5	29	11	98	37.2	125	47.3
10. I believe that natural immunity is sufficient, and I do not think I need to take the vaccine.	15	5.7	6	2.3	29	11	93	35.2	121	45.8
11. The vaccine may harm future pregnancies.	11	4.2	10	3.8	26	9.8	107	40.5	110	41.7
Scale	Min			Max			Mean			SD
	11.0			55.0			40.9			6.3

As regards perception of benefits of COVID-19 vaccination, half of the participants 50% agreed that vaccination would alleviate the precautionary measures, including lockdown, quarantine, work permit, and travel bans. Additionally, 40.9% of them agreed that COVID-19 complications are more serious in pregnant women and that the vaccine would provide protection (Table 4).

**Table 4:** Perception of benefits of COVID-19 vaccination among the participants.

Item	Strongly disagree		Disagree		Neutral		Agree		Strongly agree	
1. Vaccination would ease the precautionary measures including lockdown, quarantine, work permit and travel ban.	No.	%	No.	%	No.	%	No.	%	No.	%
	3	1.1	7	2.7	10	3.8	132	50	112	42.4
2. Complications of COVID-19 are more serious in pregnant women so the vaccine will protect me against them.	19	7.3	17	6.4	29	11	108	40.9	91	34.4
3. Vaccination decreases my chance of getting COVID-19 or its complications.	22	8.3	20	7.6	32	12.1	104	39.4	86	32.6
4. Taking the vaccine would make me less worried about catching COVID-19.	18	6.8	16	6.1	32	12.1	124	47	74	28
Scale	Min		Max		Mean		Mode		SD	
	6.0		20.0		15.8		19.0		3.4	

Reasons for receiving COVID-19 vaccination were variable, less than half of the participants 45.8%, 45.8%, and 41.3%, respectively agreed that they would only take the COVID-19 vaccine if it was made mandatory and widely accepted by the public, or if they were provided with adequate information about it (Table 5).

**Table 5:** Reasons for receiving COVID-19 vaccination among the participants.

Item	Strongly disagree		Disagree		Neutral		Agree		Strongly agree	
1. I will only take the COVID-19 vaccine if it is made mandatory.	No.	%	No.	%	No.	%	No.	%	No.	%
	4	1.5	3	1.1	17	6.5	121	45.8	119	45.1
2. I will only take the COVID-19 vaccine if it is taken by many in the public.	3	1.1	1	0.4	27	10.2	121	45.8	112	42.5
3. I will only take the COVID-19 vaccine if I was given adequate information about it.	6	2.3	3	1.1	29	11	109	41.3	117	44.3
Scale	Min			Max			Mean			SD
Reasons	4.0			15.0			12.8			19.9

The results revealed significant differences in the perception scale scores based on the number of children a participant had (*P* = 0.026) with participants with a higher number of children were found to have higher scores. Regarding the hesitancy scale, significant differences in scores were identified based on occupation and the vaccination status variables (*P* = 0.003 and *P* = 0.033, respectively). Participants working in the health sector exhibited lower hesitancy scores compared to others while non-vaccinated participants had a higher mean score compared to vaccinated participants. The study also demonstrated significant differences in the scores of the benefits and reasons scales based on gestational age (*P* = 0.020). Participants in the second trimester had lower mean scores compared to others in both the benefits and reasons for COVID-19 vaccination scales. Furthermore, significant differences in the scores of the reasons scale based on gestational age were observed (*P* = 0.010). Participants in the second trimester had lower mean scores compared to others in both the benefits and reasons for COVID-19 vaccination scales (Table 6). The Pearson correlation coefficients revealed significant relationships among participants' perceptions of COVID-19 severity, vaccine hesitancy, perceived benefits, and reasons for vaccination (Table 7).

**Table 6:** Basic demographic characteristics of the participants by perception, hesitancy, benefits, and reasons scale.

Basic demographic characteristics of the participants by perception scale:		
Variable	Mean ± SD	*P*-value
No. of children		
0	11.2 ± 3.4	
1–3	11.3 ± 3.5	
4–6^ƿ^	13.0 ± 3.4	0.026*
7+	11.8 ± 2.5	
Basic demographic characteristics of the participants by hesitancy scale:		
Occupation		
Housewife	41.1 ± 6.2	
Working in health sector^ƿ^	37.1 ± 8.5	0.003*
Working outside health sector	41.8 ± 4.8	
Vaccination status		
None^ƿ^	44.3 ± 5.6	0.033*
One dose	42.2 ± 4.3	
Two doses	40.2 ± 6.7	
Three doses	40.5 ± 6.3	
Basic demographic characteristics of the participants by benefits scale:		
Gestational age		
1st trimester	15.9 ± 4.0	
2nd trimester^ƿ^	15.0 ± 3.7	0.020*
3rd trimester	16.3 ± 3.0	
Basic demographic characteristics of the participants by reasons scale:		
Gestational age		
1st trimester	13.6 ± 1.6	
2nd trimester^ƿ^	12.4 ± 2.1	0.010*
3rd trimester	13.0 ± 1.9	

* Significant differences.

^*ƿ*^ Significant group.

**Table 7:** Correlation between perception, hesitancy, benefits, and reasons scales.

		Perception of COVID-19 severity	Hesitancy to vaccine	Benefits	Reasons
Perception of COVID-19 Severity	Pearson Correlation	1	.276**	.133*	.184**
	Sig. (2-tailed)		.000	.031	.003
Hesitancy to vaccine	Pearson Correlation	.276**	1	.270**	.389**
	Sig. (2-tailed)	.000		.000	.000
Benefits	Pearson Correlation	.133*	.270**	1	.333**
	Sig. (2-tailed)	.031	.000		.000
Reasons	Pearson Correlation	.184**	.389**	.333**	1
	Sig. (2-tailed)	.003	.000	.000	

* Correlation is significant at the 0.05 level (2-tailed).

** Correlation is significant at the 0.01 level (2-tailed).

## 4 Discussion

In the present study, approximately half (48.9%) of the participants agreed that the likelihood of contracting a COVID-19 infection is low in Saudi Arabia. This perception aligns with the reported decrease in COVID-19 cases in the country from 13.7% to 10.9%. These reductions reflect the effectiveness of the implemented prevention strategies (3, 36).

The most reported reasons for hesitancy to receive COVID-19 vaccination among the study participants were their concerns about the vaccine safety, side effects and effectiveness during pregnancy. These findings are consistent with a study conducted by Goncu Ayhan et al. (37), which aimed to assess acceptance and hesitancy toward COVID-19 vaccines among pregnant women. The study found that those who would refuse the vaccine cited a lack of data on COVID-19 vaccine safety during pregnancy and potential harm to the fetus as their main concerns. These findings can be due to the exclusion of pregnant women from clinical trials.

Half of the participants in the present study stated that vaccination would ease precautionary measures, including lockdowns, quarantine, work permits, and travel bans. Additionally, 40.9% of them believed that COVID-19 complications are more severe during pregnancy and that the vaccine would protect them. These results are in line with a study conducted to assess the attitude toward COVID-19 vaccination among pregnant and breastfeeding women in Italy, which revealed that a significant percentage of the pregnant women believed they were at a considerable risk of contracting SARS-CoV-2, emphasizing the importance of receiving the COVID-19 vaccine (38).

Less than half of the participants in the present study agreed that they would only take the COVID-19 vaccine if it was made mandatory or widely accepted by the public. These findings correspond with a study conducted in Saudi Arabia, which aimed to explore the acceptability and reluctance of pregnant and breastfeeding women to receive COVID-19 vaccination. The study reported that about half of the women stated they would receive the COVID-19 vaccine if it were recommended for pregnant and breastfeeding women (39).

Significant differences in perception scores of COVID-19 severity and susceptibility were found based on the number of children, where participants with a higher number of children had higher scores on the scale. This can be explained by the heightened worry and concern of the mothers about themselves and their children. However, no such correlation was identified between COVID-19 vaccine perception scores of severity and susceptibility and the number of children (*P* > 0.05) in a study conducted by Goncu Ayhan et al. (37) in Turkey.

Furthermore, significant differences in hesitancy scale scores were observed based on occupation and COVID-19 vaccination status, with participants working in the health sector having lower scores compared to others. This could be attributed to the increased health awareness among those in the health sector regarding the importance of vaccination. This finding contrasts with a cross-sectional study that found no statistically significant relationships between COVID-19 vaccine acceptance and occupation status among the participants (39).

Moreover, participants who had not received the COVID-19 vaccine exhibited higher mean scores for hesitancy, which accounts for their decision to not get vaccinated. This is consistent with the results of an institutional-based cross-sectional study conducted by Mose and Yeshaneh (10) in an antenatal care clinic in Southwest Ethiopia, which revealed that pregnant women who exhibited hesitancy toward the influenza H1N1 vaccine displayed similar concerns regarding COVID-19 vaccination.

The results of the benefits and reasons for receiving the vaccine scales showed significant differences in scores among participants in the second trimester, who had lower mean scores compared to others. Another study found no significant relationship between gestational age and the scores of benefits and reasons for receiving the vaccine scales (39).

The study's findings highlight the vital role of healthcare providers in the vaccination decision-making process of pregnant women. These professionals are pivotal in enhancing vaccine acceptance, particularly by offering tailored health education during antenatal visits that focuses on the severity of COVID-19, the susceptibility of pregnant women to the infection, and the benefits of vaccination. The provision of printed materials outlining preventive strategies and the safety of COVID-19 vaccines can further support their efforts in improving vaccine uptake among this population.

A notable limitation of this study is the use of a self-administered survey instrument, which raises concerns regarding the risk of response bias. Additionally, the employment of non-probability sampling may restrict the generalizability of the study's results. These methodological limitations suggest that future research should employ more robust sampling techniques to enhance the representativeness and reliability of the data.

To contribute to the growing body of knowledge on the subject, further research should be undertaken in diverse settings. This research should prioritize the evaluation of the safety of COVID-19 vaccines specifically in pregnant women, addressing their unique healthcare needs. In the clinical context, counseling services should be provided to pregnant women. These sessions should address their specific concerns related to COVID-19 vaccination. By doing so, healthcare professionals can play a crucial role in enhancing awareness and acceptance of the COVID-19 vaccine among pregnant women.

The findings of the present study showed that the majority of participants perceived a low susceptibility to COVID-19 infection and expressed hesitancy toward the vaccine, with uncertainty about its effectiveness. However, they indicated a willingness to receive the COVID-19 vaccine if it was made mandatory and if they were provided with adequate information.

## Data Availability

The original contributions presented in the study are included in the article, further inquiries can be directed to the corresponding author.

## References

[1] López M Gonce A Meler E Plaza A Hernández S Martinez-Portilla RJ Coronavirus disease 2019 in pregnancy: a clinical management protocol and considerations for practice. Fetal Diagn Ther. (2020) 47:519–28. 10.1159/000508487 32535599 PMC7362587

[2] WHO. World Health Organization. Ten threats to global health in 2019; 2019 (2022). Available online at: https://www.who.int/news-room/spotlight/ten-threats-to-global-health-in-2019 (Accessed March 9, 2022).

[3] MOH. COVID-19 & vaccine FAQs. Ministry of health Saudi Arabia (2022). Available online at: https://www.moh.gov.sa/en/Ministry/HotTopics/Pages/COVID-19-Vaccine.aspx (Accessed March 31, 2022).

[4] Ellington S Strid P Tong VT Woodworth K Galang RR Zambrano LD Characteristics of women of reproductive age with laboratory-confirmed SARS-CoV-2 infection by pregnancy Status — United States, January 22–June 7, 2020. Morb Mortal Wkly Rep. (2020) 69:769–75. 10.15585/mmwr.mm6925a1 PMC7316319 32584795

[5] Weetman AP. Immunity, thyroid function and pregnancy: molecular mechanisms. Nat Rev Endocrinol. (2010) 6(6):311–8. 10.1038/nrendo.2010.46 20421883

[6] Pazos M Sperling RS Moran TM Kraus TA. The influence of pregnancy on systemic immunity. Immunol Res. (2012) 54(1–3):254–61. 10.1007/s12026-012-8303-9 22447351 PMC7091327

[7] Rasmussen SA Smulian JC Lednicky JA Wen TS Jamieson DJ. Coronavirus disease 2019 (COVID-19) and pregnancy: what obstetricians need to know. Am J Obstet Gynecol. (2020) 222(5):415–26. 10.1016/j.ajog.2020.02.017 32105680 PMC7093856

[8] Guo F Yang X. A comprehensive review of the management of pregnant women with COVID-19: useful information for obstetricians. Infect Drug Resist. (2021) 14:3363–78. 10.2147/IDR.S325496 34466003 PMC8402981

[9] Shams T Alhashemi H Madkhali A Noorelahi A Allarakia S Faden Y Comparing pregnancy outcomes between symptomatic and asymptomatic COVID-19 positive unvaccinated women: multicenter study in Saudi Arabia. J Infect Public Health. (2022) 15(8):845–52. 10.1016/j.jiph.2022.06.002 35779468 PMC9225930

[10] Mose A Yeshaneh A. COVID-19 vaccine acceptance and its associated factors among pregnant women attending antenatal care clinic in Southwest Ethiopia: institutional-based cross-sectional study. Int J Gen Med. (2021) 14:2385–95. 10.2147/IJGM.S314346 34135622 PMC8197585

[11] Liu Y Chen H Tang K Guo Y. Clinical manifestations and outcome of SARS CoV-2 infection during pregnancy. J Infect. (2020) 4453(20):30109–2. 10.1016/j.jinf.2020.02.028 PMC7133645 32145216

[12] Wang CL Liu YY Wu CH Wang CY Wang CH Long CY. Impact of COVID-19 on pregnancy. Int J Med Sci. (2021) 18(3):763–7. 10.7150/ijms.49923 33437211 PMC7797535

[13] Chen H Guo J Wang C. Clinical characteristics and intrauterine vertical transmission potential of COVID-19 infection in nine pregnant women: a retrospective review of medical records. Lancet. (2020) 395(10226):809–15. 10.1016/S0140-6736(20)30360-3 32151335 PMC7159281

[14] Breslin N Baptiste C Miller R. COVID-19 in pregnancy: early lessons. Am J Obstet Gynecol. (2020) 2(2):100111. 10.1016/j.ajogmf.2020.100111 PMC7271091 32518902

[15] Lokken EM Walker CL Delaney S. Clinical characteristics of 46 pregnant women with a severe acute respiratory syndrome coronavirus 2 infection in Washington state. Am J Obstet Gynecol. (2020) 223(6):911.e1–911.e14. 10.1016/j.ajog.2020.05.031 32439389 PMC7234933

[16] Huang C Wang Y Li X Ren L Zhao J Hu Y. Clinical features of patients infected with 2019 novel coronavirus in Wuhan, China. Lancet. (2020) 395(10223):497–506. 10.1016/S0140-6736(20)30183-5 31986264 PMC7159299

[17] Ramos IF Guardino CM Mansolf M Glynn LM Sandman CA Hobel CJ. Pregnancy anxiety predicts shorter gestation in Latina and non-Latina white women: the role of placental corticotrophin-releasing hormone. Psychoneuroendocrinology. (2019) 99:166–73. 10.1016/j.psyneuen.2018.09.008 30245329 PMC6231951

[18] Hunt B Retter A McClintock C. Practical Guidance for the Prevention of Thrombosis and Management of Coagulopathy and Disseminated Intravascular Coagulation of Patients Infected With COVID-19. Llanwrda, UK: Thrombosis UK (2020).

[19] Zambrano LD Ellington S Strid P. Update: characteristics of symptomatic women of reproductive age with laboratory-confirmed SARS-CoV-2 infection by pregnancy status — United States. MMWR Morb Mortal Wkly Rep. (2020) 69:1641–7. 10.15585/mmwr.mm6944e3 33151921 PMC7643892

[20] Mendoza M Garcia-Ruiz I Maiz N Rodo C Garcia-Manau P Serrano B Preeclampsia-like syndrome induced by severe COVID-19: a prospective observational study. BJOG. (2020) 127(11):1374–80. 10.1111/1471-0528.16339 32479682 PMC7300912

[21] Allotey J Stallings E Bonet M Yap M Chatterjee S Kew T Clinical manifestations, risk factors, and maternal and perinatal outcomes of coronavirus disease 2019 in pregnancy: living systematic review and meta-analysis. Br Med J. (2020) 370:m3320. 10.1136/bmj.m3320 32873575 PMC7459193

[22] Pereira A Cruz-Melguizo S Adrien M Fuentes L Marin E Perez-Medina T. Clinical course of coronavirus disease-2019 in pregnancy. Acta Obstet Gynecol Scand. (2020) 99(7):839–47. 10.1111/aogs.13921 32441332 PMC7280597

[23] Blitz MJ Rochelson B Meirowitz N Prasannan L Rafael TJ. Maternal mortality among women with coronavirus disease 2019. Am J Obstetrics Gynecol. (2019) 223(4):595–9. 10.1016/j.ajog.2020.06.020 PMC7294262 32553910

[24] Xu L Yang Q Shi H Lei S Liu X Zhu Y Clinical presentations and outcomes of SARS-CoV-2 infected pneumonia in pregnant women and health status of their neonates. Science Bulletin. (2020) 65(18):1537–42. 10.1016/j.scib.2020.04.040 32346493 PMC7186128

[25] Ceulemans M Foulon V Panchaud A Winterfeld U Pomar L Lambelet V Vaccine willingness and impact of the COVID-19 pandemic on women’s perinatal experiences and practices—a multinational, cross-sectional study covering the first wave of the pandemic. Int J Environ Res Public Health. (2021) 18(7):3367. 10.3390/ijerph18073367 33805097 PMC8038007

[26] WHO. Strategy to achieve global COVID-19 vaccination by mid-2022. WHO World Health Organization (2021). Available online at: https://www.who.int/publications/m/item/strategy-to-achieve-global-covid-19-vaccination-by-mid-2022 April 2022 (Accessed May 31, 2022).

[27] Borges MASB Florentino PTV Cerqueira-Silva T Carvalho LF Oliveira VA Aguilar GMO Factors associated with COVID-19 vaccination among pregnant women in rio De janeiro city, Brazil. Sci Rep. (2023) 13:18235. 10.1038/s41598-023-44370-6 37880238 PMC10600223

[28] Nguyen L Hoang M Nguyen L Ninh L Nguyen H Nguyen A Acceptance and willingness to pay for COVID-19 vaccines among pregnant women in Vietnam. Trop Med Int Health. (2021) 26(10):1303–13. 10.1111/tmi.13666 34370375 PMC8447150

[29] Kilich E Dada S Francis MR Tazare J Chico RM Paterson P Factors that influence vaccination decision-making among pregnant women: a systematic review and meta-analysis. PLoS One. (2020) 15(7 July 2020):1–28. 10.1371/journal.pone.0234827 PMC7347125 32645112

[30] Glanz JM Wagner NM Narwaney KJ Kraus CR Shoup JA Xu S Web-based social Media intervention to increase vaccine acceptance: a randomized controlled trial. Paediatrics. (2017) 140(6):e20171117. 10.1542/peds.2017-1117 PMC8574135 29109107

[31] Qin Y Zhou R Wu Q Huang X Chen X Wang W The effect of nursing participation in the design of a critical care information system: a case study in a Chinese hospital. BMC. (2017) 17(1):165. 10.1186/s12911-017-0569-3 PMC5719644 29212480

[32] Bichovsky Y Klein M Brotfain E. A positive cumulative fluid balance in critically ill patients: is it really harm for everybody? Kardiol Pol. (2019) 77(12):1121–2. 10.33963/KP.15111 31855195

[33] Liu J Cao Y Xu C Zhou C Wei W Yuan J Midwifery and nursing strategies to protect against COVID-19 during the third trimester of pregnancy. Midwifery. (2021) 92:102876. 10.1016/j.midw.2020.102876 33220602 PMC7834539

[34] Samannodi M. COVID-19 vaccine acceptability among women who are pregnant or planning for pregnancy in Saudi Arabia: a cross-sectional study. Patient Prefer Adherence. (2021) 15:2609–18. 10.2147/PPA.S338932 34866902 PMC8633708

[35] Almaghaslah D Alsayari A Kandasamy G Vasudevan R. COVID-19 vaccine hesitancy among young adults in Saudi Arabia: a cross-sectional web-based study. Vaccines. (2021) 9(4):330. 10.3390/vaccines9040330 33915890 PMC8067112

[36] WHO. Weekly operational update on COVID-19 - 10 April 2022 (2022). Available onlin at: https://www.who.int/publications/m/item/weekly-epidemiological-update-on-covid-19—12-april-2022 (Accessed May 31, 2022).

[37] Goncu Ayhan S Oluklu D Atalay A Menekse Beser D Tanacan A Moraloglu Tekin O COVID-19 vaccine acceptance in pregnant women. Int J Gynaecol Obstet. (2021) 154(2):291–6. 10.1002/ijgo.13713 33872386 PMC9087778

[38] Carbone L Mappa I Sirico A Di Girolamo R Saccone G Di Mascio D Pregnant women’s perspectives on severe acute respiratory syndrome coronavirus 2 vaccine. Am J Obstet Gynecol MFM. (2021) 3(4):100352. 10.1016/j.ajogmf.2021.100352 33771762 PMC7985679

[39] Alshahrani SM Alotaibi A Almajed E Alotaibi A Alotaibi K Albisher S Pregnant and breastfeeding women’s attitudes and fears regarding COVID-19 vaccination: a nationwide cross-sectional study in Saudi Arabia. Int J Womens Health. (2022) 14:1629–39. 10.2147/ijwh.s387169 36457719 PMC9707376

